# The burden of musculoskeletal disorders in Belgium: a national population-based study

**DOI:** 10.1093/eurpub/ckac129.557

**Published:** 2022-10-25

**Authors:** V Gorasso, J Van der Heyden, R De Pauw, I Pelgrims, K De Ridder, S Vandevijvere, S Vansteelandt, B Vaes, D De Smedt, B Devleesschauwer

**Affiliations:** 1 Epidemiology and Public Health, Sciensano, Brussels, Belgium; 2 Public Health and Primary Care, Ghent University, Ghent, Belgium; 3 Rehabilitation Sciences, Ghent University, Ghent, Belgium; 4 Chemical and Physical Health Risks, Sciensano, Brussels, Belgium; 5 Applied Mathematics and Statistics, Ghent University, Ghent, Belgium; 6 Medical Statistics, LSHTM, London, UK; 7 Public Health and Primary Care, KU Leuven, Leuven, Belgium; 8 Veterinary Public Health and Food Safety, Ghent University, Ghent, Belgium

## Abstract

**Background:**

According to WHO, approximately one in three people worldwide live with a chronic, painful musculoskeletal (MSK) disorder. Low back pain (LBP), neck pain (NKP), osteoarthritis (OST) and rheumatoid arthritis (RHE) are among the most disabling MSK disorders. According to the Global Burden of Disease (GBD) study, LBP was the leading global cause in terms of years lived with disability and OST showed an increase in prevalence and is predicted to be one of the leading future causes. Our study aimed to analyse the burden of these MSK disorders in Belgium, providing a summary of morbidity and mortality outcomes from 2013 to 2018.

**Methods:**

Prevalence and disability-adjusted life years (DALY) were computed using data from the Belgian health interview surveys from 2013 and 2018, the INTEGO database (Belgian registration network for general practitioners) and GBD study 2019. Mortality data was retrieved from the Belgian statistical office for people dying from RHE. Following GBD methodology, LBP, NKP and OST were assumed to not generate any deaths.

**Results:**

The prevalence of MSK disorders increased from 2013 to 2018 with OST being the disorder with the highest number of cases (1.7 million cases in 2018). The burden was higher in women and the gender disparities increased with age. Women died also more frequently due to RHE compared to men. In total MSK disorders contributed to 180,746 comorbidity-adjusted DALYs for female and 116,063 comorbidity-adjusted DALYs for men in 2018, with LBP being the largest contributor (140,031 DALY).

**Conclusions:**

The burden of MSK disorders has increased over the years. In 2018, 2.5 million Belgians were affected by at least one MSK disorder that resulted in almost 300,000 DALY. Our study provides valuable information of a part of the health burden that is known to have a great impact on the total burden of disease but that is sometimes disregarded by public health institutions.

**Key messages:**

• MSK disorders represent a major health problem in Belgium.

• Acting on risk factors associated to these disorders is crucial to mitigate their burden.

